# Sphingosine-1-Phosphate Modulation in Neurological Disorders: Insights from MS and Stroke

**DOI:** 10.3390/brainsci15050436

**Published:** 2025-04-24

**Authors:** Briana Maktabi, Faheem Shehjar, Zachary Senger, Logan Kountz, Syed Hasan, Kenan Maaieh, Kylee Hoersten, Jovana Duric, Zahoor A. Shah

**Affiliations:** 1 Department of Pharmacology and Experimental Therapeutics, College of Pharmacy and Pharmaceutical Sciences, Toledo, OH 43614, USA; 2 Department of Medicinal and Biological Chemistry, College of Pharmacy and Pharmaceutical Sciences, Toledo, OH 43614, USA; 3 Department of Pharmacy Practice, College of Pharmacy and Pharmaceutical Sciences, Toledo, OH 43614, USA

**Keywords:** multiple sclerosis, stroke, sphingosine-1-phosphate receptor, neuroinflammation

## Abstract

Multiple sclerosis is a chronic autoimmune disease in which the immune system attacks the protective sheath or myelin that covers nerve fibers, impacting the brain’s ability to communicate with other areas of the body. This abnormal immune response recruits inflammatory substances, which appear as lesions on the brain and spinal cord. A stroke is characterized by a sudden impairment of neurological function resulting from the loss or restriction of blood flow due to acute damage to a localized area of the central nervous system, including the brain, retina, or spinal cord. While strokes, both ischemic and hemorrhagic, are different in terms of their pathogenesis to MS, mechanisms such as neuroinflammation and neurodegeneration are common denominators among these conditions. Recent studies highlight the involvement of the sphingosine-1-phosphate receptor in the treatment of strokes and how fingolimod, an S1P receptor modulator employed in MS treatment, may play a role in the treatment of stroke-like symptoms. This review aims to explore the potential link between stroke and MS, providing a comprehensive analysis of the existing evidence. It will also shed light on the role of S1P receptors in the pathophysiology of stroke, offering insights into their mechanistic contributions. Furthermore, the review will examine recent studies investigating the therapeutic potential of the S1P modulator, fingolimod, in acute stroke patients, highlighting its efficacy and potential clinical applications. Through this multifaceted approach, we hope to contribute to the development of a deeper understanding of these interconnected neurological conditions and their treatment strategies.

## 1. Introduction

Multiple sclerosis (MS) and stroke are two distinct neurological disorders that, despite their differences in terms of etiology and pathophysiology, share several overlapping mechanisms, particularly in terms of neuroinflammation and neurodegeneration. MS involves a chronic inflammatory response that leads to progressive neurodegeneration, whereas stroke induces an acute inflammatory cascade that exacerbates neuronal damage. The intersection between these mechanisms raises important questions about potential therapeutic overlaps and the broader implications of targeting shared pathways in both diseases.

Recent studies have identified the sphingosine-1-phosphate (S1P) receptor as a significant player in the treatment of MS, with promising implications for stroke therapy. S1P receptor modulators, such as fingolimod, have been widely used in the treatment of MS due to their ability to modulate immune responses, reduce neuroinflammation, and potentially provide neuroprotection. Emerging evidence suggests that these modulators may also confer benefits in acute stroke by reducing inflammatory damage, enhancing blood–brain barrier (BBB) integrity, and promoting vascular repair. This raises the intriguing possibility that therapies developed for MS could be repurposed for stroke treatment, thereby improving patient outcomes across both conditions. Additionally, stroke patients with pre-existing MS may face unique challenges due to their compromised neurovascular health, making it even more imperative to explore treatment strategies that address both neuroinflammatory and ischemic components.

This review aims to provide a comprehensive analysis of the connection between MS and stroke, exploring their shared inflammatory mechanisms, the role of S1P receptors in disease progression, and the therapeutic potential of fingolimod and other S1P receptor modulators. Furthermore, it will assess how clinical and preclinical studies have evaluated the effectiveness of S1P modulators in stroke and MS and discuss their mechanistic contributions to neuroprotection and functional recovery. By synthesizing current research and clinical studies, we seek to shed light on the translational potential of MS therapies in stroke management.

### 1.1. Multiple Sclerosis

**Figure 1:**
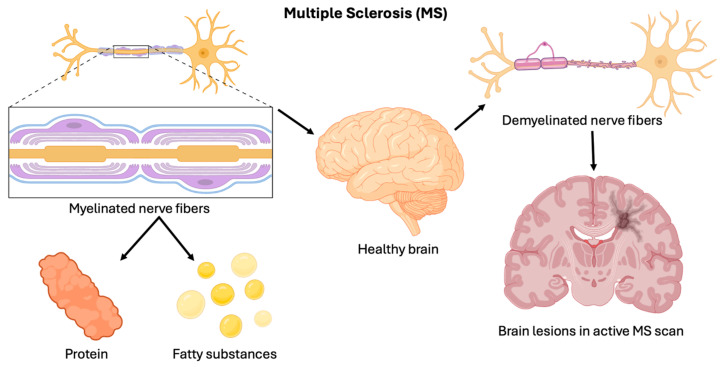
MS overview. An illustration of MS showing the difference between myelinated and de-myelinated nerve fibers. The figure highlights the role of proteins and fatty substances in myelin formation, the impact of demyelination on the brain, and the presence of brain lesions in an active MS scan.

MS is a chronic autoimmune disease in which the immune system attacks the protective sheath or myelin that covers nerve fibers (Figure 1), impacting the brain’s ability to communicate with other areas of the body [1]. Myelin is made up of protein and fatty substances. It is a protective and insulating layer that forms around nerves, including those in the brain and spinal cord. Myelin is responsible for the efficient transmission of electrical impulses between nerve cells. If myelin is damaged, these impulses slow down, leading to a wide variety of neurological symptoms [2]. This abnormal immune response recruits inflammatory substances, which appear as lesions on a brain magnetic resonance imaging (MRI) scan in active disease.

**Figure 2:**
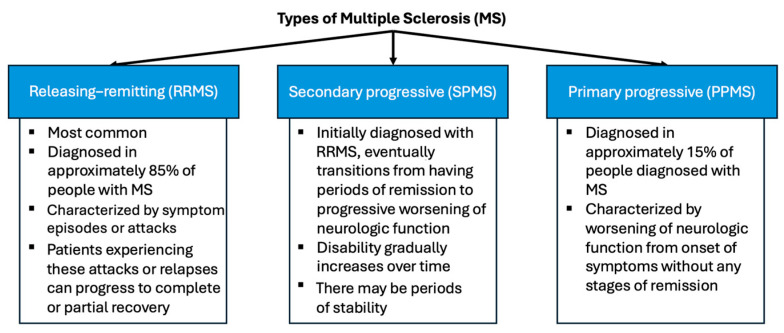
Summary of types of MS. An overview of the three main types of MS, highlighting the characteristics and progression patterns, with RRMS being the most common, SPMS developing from RRMS with increasing disability, and PPMS showing continuous neurological decline from symptom onset.

More than 2.3 million people are affected by MS worldwide, with most diagnosed between the ages of 10 and 50. There is no known cause of MS; however, scientists believe that it is a combination of environmental and genetic factors [3]. MS types can be classified into three categories (Figure 2): relapsing–remitting (RRMS), secondary progressive (SPMS), and primary progressive (PPMS). Distinguishing between the three different types of MS aids in understanding prognosis and selecting the most suitable treatment plan for each type of MS [4]. RRMS is the most common type and is initially diagnosed in approximately 85% of people with MS. It is characterized by episodes or attacks of new or worsening neurological symptoms. The patients experiencing these attacks or relapses can progress to complete or partial recovery [5]. The second type is SPMS, according to which a patient initially diagnosed with RRMS eventually transitions from having periods of remission to progressive worsening of their neurologic function over time. Disability may gradually increase over time; however, there may also be periods of stability. Lastly, approximately 15% of people with MS are diagnosed with PPMS, which is characterized by a worsening of the neurologic function from the onset of symptoms without any stages of remission [5].

An additional course of MS is known as Clinically Isolated Syndrome (CIS). It is defined as the first episode of inflammation and demyelination in the central nervous system (CNS). This episode must last for at least 24 h and is considered a characteristic of MS; however, it is not a criterion for the diagnosis of MS. When CIS is paired with noticeable lesions on an MRI, the patient is more likely to develop a second episode of neurologic symptoms and, with that, meet the criteria for diagnosis of MS [5].

Early available treatments for MS mainly suppress the immune system. Regenerative medicine-based approaches attempt to increase myelin repair by targeting endogenous progenitors or transplanting stem cells or their derivatives [6,7]. Oligodendrocytes play a crucial role in the developmental process of myelination and the restoration of CNS myelin after damage [8]. The process of remyelination involves the production of new mature oligodendrocytes, which arise from both oligodendrocyte progenitor cells (OPCs) and neural progenitors (NPs) [9].

### 1.2. Current MS Therapies

**Figure 3:**
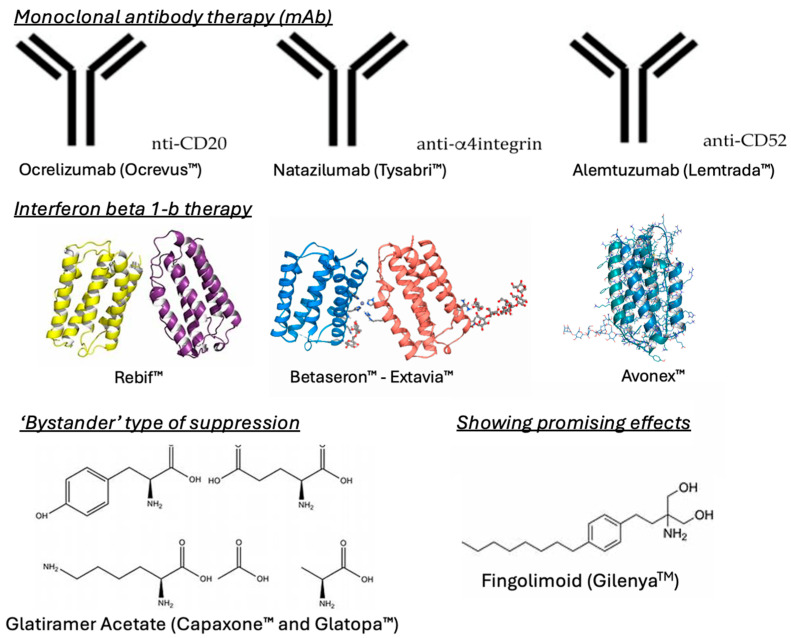
Drugs used to treat MS. An overview of drug classes used in the management of MS, including gene therapy, interferon β 1-b therapy, the ‘bystander’ type of suppression, and drugs showing promising effects.

While there are no therapies that completely cure MS, there are medications (Figure 3) that are given to patients to control the autoimmune response and subsequent lesion formation, enabling the regulation of the disease.

Many antibody therapies have been developed to control the immune response by targeting specific immune cells, such as B-cells [10]. An example of antibody therapies is ocrelizumab (Ocrevus^TM^), which offers promising clinical results, especially for the treatment of RRMS. According to the market share report, ocrelizumab is currently the most prescribed antibody therapy medication, followed by natalizumab (Tysabri^TM^). However, newer therapies, such as alemtuzumab (Lemtrada^TM^), also provide favorable efficacy in regard to the control of MS. While all these therapies are humanized monoclonal antibody treatments, they possess unique mechanisms of action. Ocrelizumab has an anti-CD20 mechanism of action and actively destroys B-cells, which in turn interrupts T-cell activation [10]. On the other hand, natalizumab halts the adhesion of leukocytes to endothelial tissues by targeting alpha-4 integrin, thus blocking them from entering the brain and progressing the disease [11]. Lastly, alemtuzumab is an anti-CD52 therapy that targets the CD52 protein on the surface of mature lymphocytes, depleting them and preventing severe disease progression [12]. The major goal of these therapies is to specifically target immune cells and proteins involved in the progression of MS and impede them from progressing the disease.

Along with gene therapies, some of the most frequently prescribed medications for the control of MS include those of the interferon β 1-b class. Interferon β 1-b has been shown to reduce the expression of proinflammatory cytokines, while increasing the expression of anti-inflammatory cytokines. There are multiple FDA-approved interferon β 1-b therapies, including Rebif^TM^, Betaseron^TM^, Avonex^TM^, and Extavia^TM^. This class of medication has been shown to ameliorate lesions and ‘black-hole’ evolution, which are common characteristics of MS [13]. Further, they are very effective in combination with other therapies and effectively control RRMS [14].

Glatiramer acetate (Capaxone^TM^ and Glatopa^TM^) offers a ‘bystander’ type of suppression, wherein the drug itself cannot cross the BBB, but can induce peripheral Th2 cells to cross the BBB and reduce neuroinflammatory symptoms [15]. As proinflammatory immune cells are the major disease collaborators, glatiramer acetate effectively changes the characterization of the cells to reduce their proinflammatory effects and promote a neutral or regulatory effect in the lymphocytes. Glatiramer acetate can be injected in 20 mg and 40 mg doses and is used effectively in combination with gene therapy or interferon β1-b. Through the binding of major histocompatibility complexes, the drug can effectively target all immune antigen-presenting cells [13].

Recent studies have shown the promising effects of a chemical drug known as fingolimod (Gilenya^TM^). The structure of fingolimod was originally designed and synthesized in Japan by researchers at Kyoto University, Taito, and Yoshitomi Pharmaceutical Industries [16]. In 1995–1996, the structure of fingolimod was first described, as part of a chemical derivatization program based on the fungal metabolite, myriocin, which is also known as ISP-1 [17]. Fingolimod acts differently to classical immunosuppressants, such as corticosteroids, which sparked an interest in further investigations. Early studies in animals showed that in combination with calcineurin inhibitors, fingolimod produced a synergistic effect by inhibiting the proliferation of T-cells and prolonging organ graft survival [18]. Fingolimod was the first oral DMT approved by the FDA for relapsing forms of MS. It is an immunosuppressive drug that modulates the S1P receptor [19].

Interestingly, fingolimod activates the S1P receptor, while downregulating its expression, significantly reducing the number of aggressive lymphocytes infiltrating the BBB. Functionally, it operates as an antagonist, leading to the internalization and breakdown of S1P receptors, causing lymphocytes to be held back in lymph nodes [20], thereby inducing immunosuppression. Gilenya^TM^ has been used more frequently as it is an oral medication, unlike the infusion or injection therapies mentioned previously [21].

### 1.3. Immune System Involvement in MS

i. Adaptive Immune System:

CD4^+^ T-cells differentiate into Th1 and Th17 subtypes in response to specific cytokines like IL-12 and tumor growth factor beta TGF-β1. Th1 cells produce IFN-γ, promoting inflammation, while Th17 cells secrete IL-17, driving further inflammatory responses. Both cell types contribute significantly to the pathogenesis of MS by facilitating the breakdown of the BBB and promoting neuroinflammation. Regulatory T-cells (Tregs) play a critical role in limiting inflammation by producing inhibitory cytokines. In MS, a reduction in Treg numbers or dysfunction of their suppressive activity allows pathogenic T-cells to escape regulation, exacerbating the inflammatory process [22]. 

It has been suggested that CD8^+^ T-cells may also play a role in MS pathology, specifically through the deterioration of the CNS via the production of granzymes and perforins that induce cell death, and Fas/FasL-mediated cytotoxicity. It is believed these autoreactive T-cells may escape deletion in the thymus, the organ responsible for eliminating self-reactive T-cells that may potentially attack the body’s own tissue, to ultimately induce MS, which is arguably the most accepted theory at the start of MS pathogenesis [23]. They may also secrete IL-17 and proinflammatory lymphotoxin, as well as chemo-attractants IL-16 and IP-10, which attract CD4^+^ cells. The role of CD8^+^ T-cells is still not fully understood and requires more studies. B-cells have been found to secrete autoantibodies following differentiation, which may engage in antibody-dependent cytotoxicity (ADCC) and self-injury via the complement pathway, as well as produce proinflammatory cytokines and act as antigen-presenting cells to further promote T-cell activation. Immunoglobulins, or oligoclonal bands, also increase within MS patients, with an estimated 90–95% of MS patients possessing oligoclonal bands in their cerebrospinal fluid. This is often used to confirm a diagnosis of MS and is also a large indicator of inflammation in the CNS.

ii. Innate Immune System:

Mast cells play the main role in MS by releasing histamines and tryptases, opening the BBB to allow inflammatory cells into the CNS. Mast cells also release chymase, which, in combination with tryptase, activates matrix metalloproteinase (MMP) precursors, ultimately inducing neurodegeneration [24].

CNS-resident microglia and resident macrophages are activated during MS to produce inflammatory cytokines, chemokines, and neurotoxic products, such as tumor necrosis factor alpha (TNF-α) and reactive nitrogen/oxygen species, which result in the main process of demyelination that leads to its involvement with neurodegeneration, antigen-presenting cells, and debris clearance for the regulation of remyelination [22,25]. 

Little information is known about the role of natural killer (NK) cells in MS, but strides in research have been made and more is being discovered about them in this context. One study recognized that NK cells play an immunoregulatory role in MS patients, mainly through a subset of NK cells, called CD56^bright^CD16^−^, that have a limited cytotoxic capability, but release many cytokines within peripheral lymphoid tissues. More research is needed to fully understand the role of NK cells in MS [26].

Astrocytes express toll-like receptors (TLRs) and MHC class I and II, allowing them to play a significant role in MS pathogenesis. They may also regulate access to the BBB and influence T-cell cytokine production. It is also noted that astrocytes repair tissue and offer neuroprotection by confining inflammation via scar formations. Genetic variations may cause faults in this pathway that initiate the pathogenesis of MS [27].

## 2. Stroke

Stroke is a leading cause of disability and death worldwide, primarily resulting from an acute disruption of blood flow to the brain. This interruption may be due to vascular occlusion (ischemic stroke) or vessel rupture (hemorrhagic stroke), both of which initiate cascades of inflammation, oxidative stress, and BBB breakdown that exacerbate neurological injury [19,20,21]. While the pathophysiological mechanisms differ between ischemic and hemorrhagic stroke, both share common neuroinflammatory processes that are relevant to S1P receptor signaling [28]. In ischemic stroke, reperfusion injury leads to the release of reactive oxygen species (ROS) and pro-inflammatory cytokines, further damaging neurons and vascular structures [29]. Hemorrhagic stroke similarly activates resident microglia and recruits peripheral immune cells, leading to cytokine production and edema that impede neurological recovery [30]. Given this inflammatory milieu, targeting immune cell trafficking and endothelial integrity has emerged as a therapeutic strategy. In this context, the modulation of sphingosine-1-phosphate (S1P) receptors, especially S1P1 and S1P3, has been investigated for its ability to reduce neuroinflammation and preserve BBB function [28].

**Figure 4:**
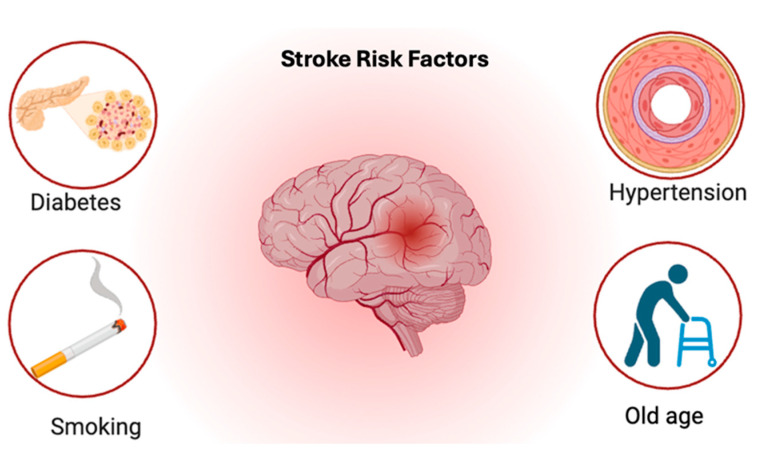
Stroke risk factors. Factors, such as diabetes, smoking, hypertension, and old age, etc., contribute to an increased likelihood of stroke by affecting blood vessel health and brain function [37].

The majority of strokes have been reported in individuals over 65 years old, indicating that old age is a significant risk factor [31]. However, strokes can also affect younger adults, particularly those with underlying risk factors (Figure 4), such as hypertension, diabetes, and smoking [32]. The etiology of stroke is multifactorial, involving a complex interplay of genetic, environmental, and lifestyle factors, contributing to the development and precipitation of cerebrovascular events [29]. Understanding the primary causes of stroke is essential for effective prevention, risk stratification, and management [29,30,31,32,33,34,35,36].

**Figure 5:**
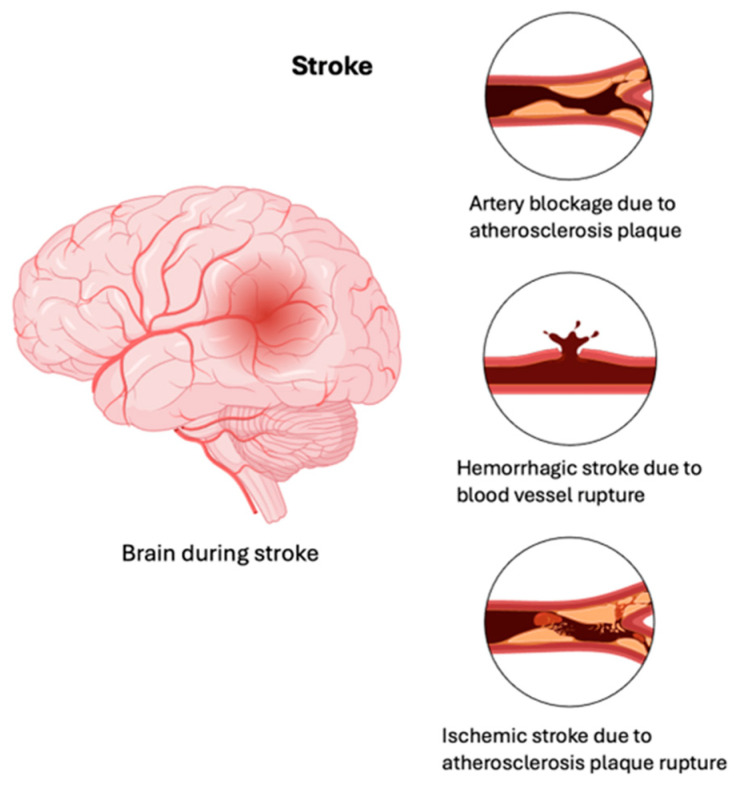
Types of stroke. A schematic representation of the brain during stroke and major types of stroke: (1) artery blockage due to atherosclerosis plaque; (2) hemorrhagic stroke due to blood vessel rupture; and (3) ischemic stroke due to atherosclerosis plaque rupture [42,43].

There are two main types of stroke, namely ischemic and hemorrhagic, each with its own distinct pathophysiological mechanisms (Figure 5).

An ischemic stroke occurs when a blood vessel supplying blood to the brain is obstructed by a blood clot or a buildup of fatty deposits called plaque. The obstruction restricts blood flow, leading to a lack of delivery of oxygen and nutrients to the brain cells supplied by the affected vessel [38]. A hemorrhagic stroke occurs when a weakened blood vessel ruptures and leaks blood into the brain (intracerebral hemorrhage; ICH) or into the space surrounding the brain (subarachnoid hemorrhage) [39]. The presence of blood within or around the brain can exert pressure on surrounding tissues, leading to damage and neurological deficits [34]. A transient ischemic attack (TIA) can be described as a minor stroke, typically lasting only a few minutes, due to temporary disruption of normal blood flow to the brain [40]. TIAs produce stroke-like symptoms, but do not cause permanent brain damage. However, TIAs may predispose a patient to an increased risk of a full-blown stroke and should prompt immediate medical evaluation and intervention [34,38,39,40,41].

### Current Stroke Therapies

The best medication or therapy a patient experiencing an ischemic stroke could receive is a tissue plasminogen activator (tPA), which is an example of thrombolytic therapy, which effectively removes blood clots and promotes proper blood flow in the brain [44]. A tPA has a narrow therapeutic window, but in the event of an acute ischemic stroke, works well within a few hours of onset to dissolve blood clots and restore circulation. Blood thinners, such as aspirin, anticoagulants, such as heparin, and blood pressure medications effectively reduce the strain on brain vasculature and are recommended to promote blood flow. Aside from blood pressure medication, the opposite approach should be taken in regard to a hemorrhagic stroke event, where the patient is actively bleeding from the brain tissue and, thus, should cease taking any type of blood thinner or anticoagulant. Blood pressure medications are usually given to manage the blood flow to the brain in a hemorrhagic stroke event, and a CT scan is performed to aid in identifying the source of the bleed. From here, in a clinical setting, interventional surgical techniques can be employed to embolize or close ruptures in the vasculature and reduce bleeding. Furthermore, clinicians may administer vitamin K to help with blood coagulation. In more severe cases, surgical procedures may be performed, such as a craniotomy, to remove the damaged portion of brain tissue and relieve any intracranial pressure from the hemorrhage and resulting lymphocyte infiltration [39]. Currently, managing blood pressure, reversing the role of blood thinners, and surgical interventions are the best methods for managing hemorrhagic stroke conditions.

## 3. S1P Receptors as Targets for MS and Stroke

S1P, a naturally occurring lipid mediator, acts on multiple organs in the body by influencing various biological actions. Thus far, there are very few studies that outline the role that S1P receptors play in regard to immune function, vascular integrity, and neurobiology. While the underlying mechanism is still unknown, several studies have indicated that S1P receptors play a role regarding the vascular integrity of the BBB [45]. When considering immune function, S1Ps have been reported to play a role in immune cell trafficking, immune signaling, and immune cell activation [46].

The actions of S1P are mainly mediated by five specific G-protein coupled receptors (S1P_1–5_) [47]. S1PR_1_ was the first S1P receptor to be identified [48]. S1PR_1_ has been shown to promote egress from lymphoid tissue [49]. This egress of lymphoid organ tissue is involved in BBB integrity and neuroprotection. S1PR_2_, which is found in the CNS, was found to stimulate inflammatory marker expression during leukocyte trafficking, as well as increase vascular permeability [50,51,52]. S1PR_3_ has been shown to play a role in vascular function and neuroinflammation, influencing stroke outcomes [53]. S1PR_4_ plays a role in autoimmunity, potentially relevant to multiple sclerosis [54]. S1PR_5_ is thought to play a key role in myelination; however, studies on S1PR_5_-null mice indicate that myelination develops normally even in the absence of S1PR_5_ [55,56].

In vitro experiments have revealed that S1P signaling activates morphological changes associated with neurite extension and retractions and growth cone formation in neurons [57]. The expression of S1P receptors can vary and have opposing functions in regard to other cell types. S1P also plays a role in the regulation of synaptic activity. Neuronal viability and neurogenesis, as well as the regulation of synaptic activity, are likely key factors that may support the notion that these receptors are drug targets in MS. Excessive levels of S1P have also been associated with inflammatory sites in the pathology of MS [58].

As previously mentioned, the S1P receptor has been identified as a major therapeutic target for the investigation and treatment of multiple sclerosis. Currently there are three well-known and well-studied drugs that are used to modulate the S1P receptor’s function for the treatment of multiple sclerosis. These drugs are siponimod (Arzerra^TM^), ozanimod (Zetia^TM^), and ponesimod (Ponaris^TM^) and they all share the goal of preventing lymphocyte migration into the circulatory system and, further, into the central nervous system (CNS) [43]. By modulating the effects of the S1P receptor(s), lymphocytes are prevented from entering the bloodstream, which consequently means that they will not enter the CNS and cause exacerbated neuroinflammatory effects. While each of these drugs share the same end goal, they differ in regard to specificity and metabolism. For example, siponimod and ozanimod are selective for both S1P receptors 1 and 5, whereas ponesimod is only selective for S1P receptor 1 [44]. Further in terms of metabolism, siponimod and ponesimod do not require further metabolism to exert their effects, whereas ozanimod must be metabolized into its active metabolite to exert effects on S1P1/5 receptors [45]. Clinically, siponimod and ozanimod introduce a higher risk of atrioventricular blockage compared to that of ponesimod [46]. Ponesimod also boasts a shorter half-life, leading to a quicker reversibility of its effects and a more favorable risk/benefit profile.

The first successful drug to target S1P, FTY720 (Fingolimod, Gilenya, Novartis), is a structural analogue of sphingosine [59]. Fingolimod is currently used for the treatment of MS, but clinical trials are also underway to investigate its use in acute stroke. However, the S1P receptors that mediate these drug actions in each disease type remain unclear. There is evidence that fingolimod acts directly on the CNS. It crosses the BBB and is rapidly phosphorylated into its active form, FTY720-P [60]. This leads to an increased brain concentration of both forms of fingolimod relative to the levels in the blood. All four of the receptors that fingolimod acts on are expressed at various degrees on microglia, astrocytes, neurons, and oligodendrocytes [61]. Phosphorylated fingolimod (pFTY720) relies on S1PR_3_ to protect astrocytes from oxygen–glucose deprivation-induced neuroinflammation by inhibiting the TLR2/3-PI3K-NFkB signaling pathway [28,53].

While strokes, both ischemic and hemorrhagic, are different in terms of their pathogenesis than MS, the mechanisms of action, such as neuroinflammation and neurodegeneration, are common denominators among these conditions. Recent studies highlight the involvement of the S1P receptor in the treatment of stroke and how Gilenya^TM^ may be beneficial in individuals who are experiencing stroke-like symptoms after multiple treatments using certain gene therapies [62,63,64,65].

## 4. Fingolimod Mechanism of Action

**Figure 6:**
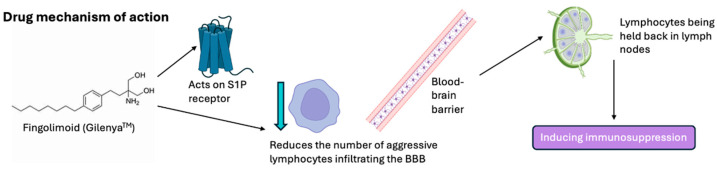
Fingolimod mechanism of action. Fingolimod, a spingosine-1-phosphate (S1P) receptor modulator, reduces the number of aggressive lymphocytes infiltrating the BBB, a result of which lymphocytes are held back in lymph nodes preventing their migration into the central nervous system (CNS), reducing neuroinflammation and demyelination associated with MS.

Given the immune response associated with ischemia, fingolimod’s immunomodulatory effects may play a role in lessening the extent of tissue injury associated with stroke. Consequently, it may also play a role in long-term rehabilitation. The process is thought to occur via S1P_1_ and S1P_3_ (Figure 6). When fingolimod binds to the S1P1 receptor on lymphocytes, it leads to receptor breakdown, interrupting lymphocyte recirculation [49]. This reduction in lymphocyte infiltration, along with fingolimod’s suppression of proinflammatory cytokine production, such as IL-1β, IFN-γ and TNF-α, aid in blocking the neurotoxic effects of the immune system on the CNS, reducing secondary ischemic damage [66]. In a middle cerebral artery occlusion (MCAO) model of ischemia, fingolimod reduced the downregulation of S1P1, which reduced the lesion size and improved the clinical outcomes [67].

Fingolimod may also have a protective effect on microvascular function via various mechanisms. Theories suggest that fingolimod promotes the release of the granulocyte and macrophage colony-stimulating factor (GM-CSF), which offers protection from proinflammatory cytokine-induced cell death [68]. GM-CSF prevents the expression of adhesion molecules, such as intercellular adhesion molecule-1 (ICAM-1) [69]. The prevention of ICAM-1 leads to the inhibition of leukocyte adhesion, reducing inflammation, platelet activation and, therefore, the risk of thrombosis [70]. Furthermore, fingolimod’s activity on S1P1 receptors on endothelial cells may contribute to maintaining the integrity of the BBB [71].

Therefore, these therapeutic effects may be attributed to fingolimod’s anti-inflammatory properties and possible protection of the vasculature rather than its direct effect on neurons. However, recent evidence suggests a possible direct neuroprotective effect via fingolimod’s binding to S1P1 and S1P3, preventing cell apoptosis and further ischemic injury [72].

## 5. Ongoing Clinical Trials

Preclinical studies of fingolimod in stroke have shown inconsistent efficacy outcomes, especially during most studies implementing a short treatment course involving the drug [73]. A recent animal study evaluating the impact of fingolimod on Treg function post-ischemia, pointed to the potential for a dual effect on these cell types [74]. In this study, investigators attributed fingolimod’s lack of a consistent benefit in these models to this dual effect on T-cells, specifically its ability to improve the suppressive function of Treg cells, while also increasing the resistance of T-cells to this produced suppression.

However, newer clinical trials have been published evaluating the potential therapeutic benefit of fingolimod in acute ischemic stroke (AIS).

Published in 2019, Lianto [75] and colleagues aimed to investigate the efficacy and safety of fingolimod in combination with intravenous thrombolytic alteplase in patients with AIS. Patients >18 years of age with symptoms of a neurological deficit lasting longer than 30 min and a Rankin scale (mRS) score of <1, indicating no obvious disability, were included in the study. The major exclusion criteria included any diseases, recent surgeries with significant trauma, or treatment with medications that may increase the risk of bleeding. All of the randomized patients received a bolus dose of alteplase (0.9 mg/kg), followed by an intravenous infusion. The intervention group received fingolimod 0.5 mg oral capsules once daily, with the first dose administered after alteplase on day 1 for three consecutive days. Any additional medications indicated in ischemic stroke, such as antiplatelets and anti-lipids, were administered 24 h after alteplase administration. National Institutes of Health Stroke Scale (NIHSS) scores were obtained at the baseline, followed by 14 and 90 days post-treatment. The mRS score and Barthel (BI) index, in which a higher score indicates independence when performing activities of daily living, were also obtained 14 and 90 days post-treatment. Safety outcomes and adverse effects were also recorded.

Of the 90 patients included, 45 received the fingolimod post-thrombolytic therapy. While no significant difference was observed in the NIHSS, mRS, and BI scores at 14 days post-treatment, the NIHSS and mRS scores were significantly lower and the BI index was significantly higher in the fingolimod group at 90 days post-treatment (*p* < 0.05). In addition, differences in CD4 + T, CD8 + T, CD19 + B, and CD56 + natural killer cell counts and the MRI scans among the two groups were also assessed. Confirming its effects on leukocyte sequestration, a reduction in these lymphocyte counts was observed 24 h after fingolimod administration. The MRI scan comparisons showed a smaller infarct volume in the combination treatment group in comparison to the alteplase group at 1 and 7 days post-thrombolytic therapy. The major adverse events reported included gastrointestinal bleeding and suspected pulmonary and urinary tract infections. However, the differences between the groups were not statistically significant. No hemorrhagic transformation was observed in the combination treatment group. This effect of fingolimod is likely attributable to its action on S1P1 receptors, rather than its effect on lymphocyte sequestration. This is evidenced by a preclinical study conducted on a mouse model exhibiting a positive effect on preventing hemorrhagic transformation in lymphocyte-deficient mice [76].

While the previous clinical trial evaluated the outcomes in AIS with the co-administration of fingolimod and thrombolytic therapy within the recommended 4.5 h window post-stroke onset, Tian [77] and colleagues evaluated the effect of fingolimod on the efficacy of thrombolytic therapy when administered outside of this recommended therapeutic window.

This was a prospective, multicenter, randomized, open-label, blinded endpoint clinical trial, published in 2018. The patients were >18 years of age, presenting with AIS at 4.5 to 6 h from symptom onset, with a baseline NIHSS > 4, a perfusion computed tomography (PCT)-defined mismatch ratio >1.2, evidence of occlusion on head and neck computed tomographic angiography (CTA) scans, and no patients with contraindications to alteplase were included. Patients were randomized to receive alteplase, followed by fingolimod or alteplase alone, at the doses reported in a previous study. The NIHSS and mRS scores were evaluated at the baseline, 24 h, upon discharge, and 90 days post-treatment. Lymphocyte counts and follow-up imaging were also obtained.

Of the 46 patients enrolled in the trial, 23 received fingolimod and alteplase. Patients in the combination group showed improvement in their NIHSS and mRS scores at 24 h post-treatment (*p* = 0.004, *p* = 0.037, respectively). Furthermore, a reduction in the size of the perfusion lesion, as well as the inhibition of infarct expansion, were observed with the addition of fingolimod (*p* < 0.001). An increased rate of asymptomatic ICH was observed in the combination group at 24 h. However, there was no significant difference in the rate of symptomatic ICH between the groups. The results on the exploratory outcome of a shift in anterograde and retrograde reperfusion favored the group receiving fingolimod. Signs of infection did not differ significantly between the two groups. Major adverse events reported in the combination group included two incidences of atrial fibrillation, which resolved with the administration of amiodarone, and two incidences of thrombocytopenia, which resolved without intervention.

In 2014, the clinical effects of fingolimod in ICH were evaluated in a 2-arm, evaluator-blinded, proof-of-concept study [78]. The authors concluded that administering fingolimod at a dose of 0.5 mg, orally, for three consecutive days, started no later than 72 h after symptom onset, could modulate brain inflammation and reduce perihematomal edema post-ICH. Patients showed improved outcomes in terms of regaining consciousness, mobility, and returning to baseline function. Consequently, fingolimod was found to lessen neurologic deficits and aid in early recovery. However, Diaz and colleagues note weaker evidence in the literature pointing to a beneficial effect of fingolimod in ICH. Therefore, they aimed to evaluate the impact of fingolimod in a mouse model of ICH using a study with sufficient statistical power in comparison to previously published studies in this area [79]. Interestingly, in this recent animal study, treatment with fingolimod did not demonstrate an improvement in the lesion size or behavioral outcomes, while maintaining its effect on reducing circulating lymphocyte counts. The authors concluded that these results do not necessarily rule out the potential effectiveness of S1P receptor modulators in ischemic and hemorrhagic stroke. Rather, future animal and clinical studies alike are needed to investigate the use of fingolimod in different populations and at different doses and in different treatment courses.

By modulating the immune response and preventing lymphocyte migration, fingolimod could help mitigate secondary brain injury following stroke, ultimately assisting with recovery and potentially improving long-term patient outcomes. However, given the diversity of the results, more studies are needed to accumulate robust evidence and fully understand its role in stroke management and its impact on the chronic disease course beyond acute treatment.

## 6. Limitations

Despite the promising findings discussed in this review, several limitations must be acknowledged. One major limitation is the lack of large-scale, randomized clinical trials directly comparing the effects of S1P receptor modulators, such as fingolimod, in both MS and stroke populations. Most existing studies are preclinical or involve small patient cohorts, limiting the generalizability of the findings to broader populations. Additionally, variations in study design, outcome measures, and treatment regimens create inconsistencies and make it challenging to draw definitive conclusions regarding the efficacy and safety of these therapies across different neurological conditions. The heterogeneity in disease presentation and progression in both MS and stroke further complicates the ability to establish standardized treatment approaches, necessitating more rigorous investigations.

Another critical limitation is the safety profile of fingolimod, particularly its immunosuppressive effects, which can lead to pronounced lymphopenia and increased susceptibility to infections. This necessitates careful patient selection and monitoring, especially in populations with pre-existing conditions that may predispose them to infections. Additionally, fingolimod has been associated with adverse cardiac events, including bradycardia and hypertension, as well as macular edema and elevated liver enzymes, requiring comprehensive pre-treatment screening and ongoing surveillance during therapy. While next-generation S1P receptor modulators are being developed to mitigate these risks, their long-term safety and efficacy remain inadequately characterized.

Furthermore, translational challenges exist between preclinical findings and clinical outcomes. While animal models have demonstrated the benefits of fingolimod in reducing infarct size and improving neurological outcomes post-stroke, these results have not consistently translated into significant clinical improvements in human trials. Differences in disease models, dosing regimens, and patient variability contribute to these discrepancies. Additionally, the long-term effects of fingolimod therapy remain underexplored, with concerns regarding potential off-target effects, such as progressive multifocal leukoencephalopathy (PML) and other rare opportunistic infections. Addressing these limitations through well-designed clinical trials and mechanistic studies will be essential in determining the viability of S1P receptor-targeting therapies for broader neurological applications.

## 7. Future Prospects and Conclusions

In conclusion, there may not be a direct link between S1P receptors, MS, and ischemic strokes. However, it is clear that S1P receptors’ involvement in these conditions is observed through the involvement of different mechanisms. In MS, the focus is on controlling inflammation and immune cell trafficking through the use of the immunomodulatory effects of S1P receptors. On the other hand, in stroke, the role of S1P receptors is focused on mitigating the damage caused by ischemia through its neuroprotective effects. Nevertheless, with more research and data showing the connection between S1P receptors and ischemic stroke, especially with the recent use of fingolimod in the treatment of this condition, this expands upon opportunities for testing other MS-approved drugs with similar mechanisms of action, such as siponimod, ozanimod, and ponesimod. While preclinical and some clinical studies have shown promising results, further research and more robust clinical trials are needed to establish the safety and efficacy of fingolimod in stroke treatment, including its impact on hemorrhagic transformation.
